# Examination of extremely low frequency electromagnetic fields on orthodontic tooth movement in rats

**DOI:** 10.1080/13102818.2014.901669

**Published:** 2014-05-06

**Authors:** Mehmet Dogru, Veysi Akpolat, Arzum Guler Dogru, Beyza Karadede, Atilim Akkurt, M. Irfan Karadede

**Affiliations:** ^a^ Department of Orthodontics, Faculty of Dentistry, University of Dicle, Diyarbakir, Turkey; ^b^ Department of Biophysics, Medical Faculty, University of Dicle, Diyarbakir, Turkey; ^c^ Department of Peridontology, Faculty of Dentistry, University of Dicle, Diyarbakir, Turkey; ^d^ Department of Orthodontics, Faculty of Dentistry, University of Yeditepe, Istanbul, Turkey

**Keywords:** electromagnetic field, tooth movement, rat

## Abstract

The purpose of this study was to evaluate whether 50 Hz extremely low frequency electromagnetic fields (ELF-EMFs) affect the amount of orthodontic tooth movement in rats. The experiments were performed on 18 male Sprague-Dawley rats. The rats were randomly divided into three groups (*n* = 6): cage-control (Cg-Cnt) group (*n* = 6); sinusoidal electromagnetic field (SEMF) group (*n* = 6); and pulsed electromagnetic field (PEMF) group (*n* = 6). In SEMF and PEMF groups, rats were subjected to 1.5 mT EMF exposure eight hours per day for eight days. In order to obtain tooth movement, holes were drilled on the right and left maxillary central incisors of the rats at a distance 1.5–2 mm away from the gingiva and 20 g of orthodontic forces were applied to the teeth. Generated linear model for repeated measures and Bonferroni tests were used to evaluate the differences between the groups. Interactions among groups by days were found by using Pillai's trace multivariate test. The results showed that significant differences were present among the groups (*F* = 5.035; *p* = 0.03) according to the extent of tooth movement. Significant differences between the amount of tooth movements were determined especially after the fifth day and the following days six, seven and eight (*p* < 0.001). Within the limitations, according to the results of the present study, the application of ELF-EMF accelerated the orthodontic tooth movement in rats.

## Introduction

Orthodontic treatment requires long durations which depend on several factors and different studies report that prolonged applications periods may be associated with some risks such as formation of caries and decreased patient cooperation.[1,2] Thus, shortening of the treatment intervals is vital in orthodontics.

Clinicians who used an increased application force during the orthodontic treatment failed to obtain accelerated tooth movement. Thus, the effects of different methods on tooth movement such as low-level laser therapy,[3] distraction osteogenesis,[4] mechanical vibration [5] and pulsed electromagnetic fields (PEMFs) [6] have been investigated.

The results of different *in vivo* and *in vitro* studies show that the application of exogenous electromagnetic fields (EMF) affect the bone metabolism.[7,8] Studies demonstrated that EMF can regulate the osteoblast proliferation and differentiation which may lead to reduction in the loss of bone mass and accelerate the bone formation in animal models.[9] Indeed, EMF had been used for the past 25 years to approach different types of osteoporosis in both animal and clinical experiments.[10,11] Although it is known that orthodontic tooth movement is accompanied by site-specific bone remodelling,[12] limited number of studies [5,6] evaluated the effects of EMF on tooth movement. Therefore, the purpose of this study was to evaluate whether a 50 Hz extremely low frequency electromagnetic field (ELF-EMF) affects the extent of orthodontic tooth movement in rats.

## Materials and methods

The experiments were performed on 18 male Sprague-Dawley rats with initial weights of 157–226 g, aged four months at the beginning of the study, which were obtained from the Medical Science Application and Research Center of Dicle University. All rats were born from one mother and allowed free access to water and standard pelleted food diet (TAVAS Inc. Adana, Turkey) during the experimental period. The rats were randomly divided into three groups (*n* = 6): cage-control (Cg-Cnt) group (*n* = 6); sinusoidal electromagnetic field (SEMF) group (*n* = 6); and PEMF group (*n* = 6). In SEMF and PEMF groups, rats were subjected to exposure of 1.5 mT EMF for eight hours per day for eight days. The animals were kept in 14/10 hour light/dark environment at a constant temperature of 22 ± 3 °C, 45 ± 10% humidity.

**Figure 1.**
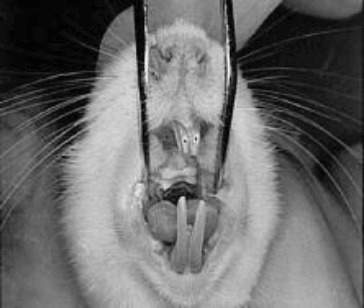
Preparation of the holes on maxillary incisor surface.

**Figure 2.**
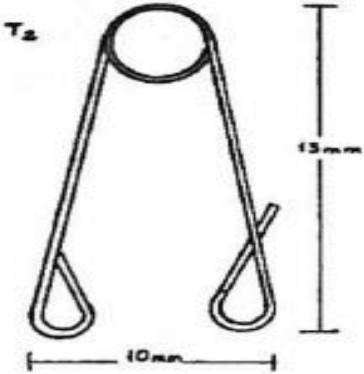
The appliance used in the study.

**Figure 3.**
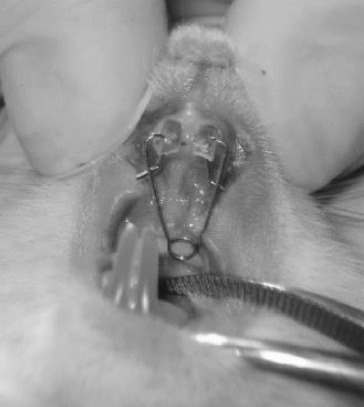
Insertion of the appliance.

**Figure 4.**
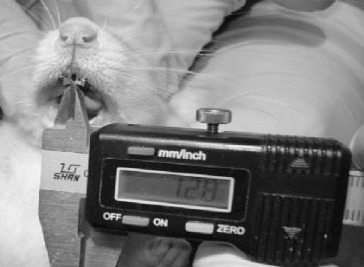
Measurement of diastema between upper incisors.

In order to obtain tooth movements, holes were drilled on the rats’ right and left maxillary central incisors 1.5–2 mm away from the gingiva (Figure 1). Following the preparation, standardized appliances bended from 0.012 inch stainless steel wire introduced by Karadede [13] were inserted into these holes (Figures 2 and 3) and 20 g of orthodontic force was applied. After the activation of the appliances, the distance between the upper incisors was measured with a digital caliper and recorded by the same investigator every 24 hours during an eight day period (Figure 4).

### Magnetic field generation and exposure of rat to magnetic field

**Figure 5.**
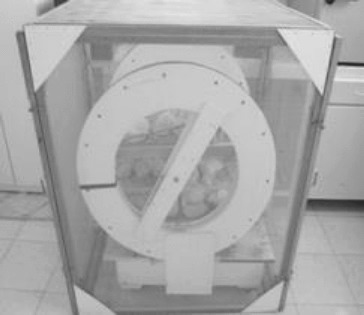
View of the specimen rat.

The SEMF was generated in a device designed by us that had two pairs of Helmholtz coils with a diameter of 70 cm in a Faraday cage (130 × 65 × 80 cm) that earthed shielding against the electric component (Figure 5).

This magnet was constructed by winding 125 turns of insulated soft copper wire with a diameter of 1.5 mm. Coils were placed vertically as facing one another. The distance between the coils was 35 cm. An alternating current (AC) produced by an AC power supply (DAYM, Turkey) was passed through the device. The current in the wires of the energized exposure solenoid was 40 A for 1.5 mT, which resulted in 50 Hz EMF. The EMF intensities were measured once per week as 1.5 mT in different 15 points of methacrylate cage with a Bell 7030 Gauss/Teslameter (F.W. BELL, Inc., SYPRIS, Orlando, FL, USA) to ensure homogeneity of the field during the course of the experiment by a person who was not involved in the animal experiment. Magnetic field measurements showed that, at the conditions of the experiment, the magnetic field exposure system produced a stable flux density of 1.5 mT and stable frequency of 50 Hz with negligible harmonics and no transients.

PEMF generated by an AC power supply (DAYM, Turkey) was passed through the device that had two pairs of Helmholtz coils. PEMF had the following attributes: a pulse width with a 1:1 mark–space ratio (50% duty cycle); a magnetic flux density of 1.5 mT; stable frequency of 50 Hz; a signal period of 20 ms (duration between pulses of 10 ms).

The static earth magnetic field was also measured with a Bell 7030 Gauss/Teslameter. The component parallel to the exposure field was 16 μT and the component perpendicular to the exposed field was 37 μT. All field measurements were performed by persons not involved in the animal experiments. Observers were not aware of the group of rats that was subjected to EMF, i.e. the whole study was done in a blind manner. No temperature differences were detected between the exposure and cage groups during the experimental treatments. The SEMF and PEMF group animals were exposed to 1.5 mT of EMF, in contrast to the Cg-Cnt group animals, for eight hours per day during eight days, placed in methacrylate boxes (33 × 32 × 15 cm). Nothing was applied to the rats in the cage control group and they completed their life cycle in the cage during the study period. The rats were free in the methacrylate cage inside the coils.

### Statistical analysis

Means and standard deviations were used to represent sets of continuous variables. The normality of the variables was analysed by Kolmogorov–Smirnov test. For the purpose of analysis, generated linear model for repeated measures followed by Bonferroni post hoc test were applied as appropriate.

Interactions among groups by days were found by using Pillai's trace multivariate test. Two-sided *p* values were considered statistically significant at *p* ≤ 0.05. Statistical analyses were carried out with the statistical packages for SPSS 15.0 for Windows (SPSS Inc., Chicago, IL, USA). A criterion level of α = 0.05 was applied for all of the used hypothesis tests.

## Results and discussion

Descriptive statistics and the results from the ‘generated linear model for repeated measures’ are shown in Table 1. The analysis of the results showed significant differences between the tested groups (*F* = 5.035; *p* = 0.030) in relation to the extent of tooth movement. There were statistically significant differences between the different treatment days in relation to the tooth movement (*F* = 4.089; *p* = 0.001). Interactions among the groups by days were found significant by the result of Pillai's trace multivariate test (*F* = 5.350; *p* = 0.01).

**Table 1.** Descriptive statistics and the results of ‘generated linear model for repeated measures’.

	Groups				
Days	SEMF	PEMF	Cg-Cnt	*F*	p**
1	1.160 ± 0.046	1.1433 ± 0.227	1.133 ± 0.430	4.089	0.001
2	1.158 ± 0.069	1.141 ± 0.351	1.12 ± 0.298		
3	1.185 ± 0.182	1.191 ± 0.061	1.15 ± 0.254		
4	1.222 ± 0.111	1.2388 ± 0.172	1.181 ± 0.118		
5	1.25 ± 0.093	1.32 ± 0.065	1.2 ± 0.120		
6	1.27 ± 0.143	1.37 ± 0.135	1.224 ± 0.081		
7	1.32 ± 0.080	1.394 ± 0.135	1.262 ± 0.075		
8	1.36 ± 0.065	1.461 ± 0.112	1.302 ± 0.098		
*F*	5.035				
p*	0.030				

Note: Interactions of group × days were found significant by the result of Pillai's trace multivariate test (*F* = 5.350; *p* = 0.01).

*According to the results of the model significant differences were found among groups (*F* = 5.035; *p* = 0.030).

**Differences among the days were found significant (*F* = 4.089; *p* = 0.001).

**Figure 6.**
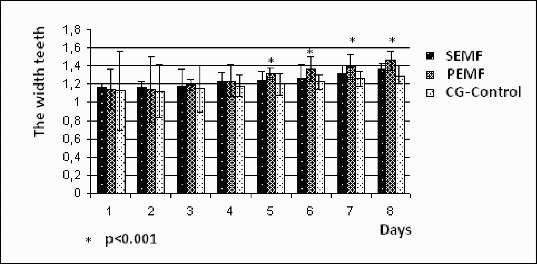
Distribution of the amount of daily tooth movement according to groups.

The distribution of the extent of daily tooth movement for SEMF, PEMF and Cg-Cnt groups is shown in Figure 6, where the differences among the groups are shown by asterisk on the figure. Significant differences between the extent of tooth movement were found especially after the fifth day followed by sixth, seventh and eighth (*p* < 0.001).

Environmental exposure to ELF-EMFs has significantly increased in developed countries as a consequence of the distribution and use of electricity.[14] The current controversial understanding of the effects of EMF on the tissues and especially on bones is one of the major challenges in medicine. The effects of EMF on cells as well as on tissues are on both cellular and transcriptional levels. The cytoskeleton of the cells is maintained by three important structures. These are microfilaments, microtubules and intermediate filaments. The cells composing tissues maintain their internal tension by cytoskeletal structures. This physiologic tensile stress in the cell is considered to be vital for the normal function of the cells. Generally when this tensile system is damaged, cells undergo apoptosis.[15,16] In addition, Blumenthal et al. [17] reported that EMFs lead to significant alteration in the cell metabolism, cytoskeleton structure as well as in its morphology. These alterations are reported to trigger the apoptosis of rat bone marrow osteoprogenitor cells and fibroblasts of the tendon in cell cultures.[17]

Tooth movement depends on the balance between the osteoblast proliferation and osteoclast activation. In order to enhance DNA, RNA and protein production in cell cultures, the potential beneficial effects of EMF can be taken into account also in clinical applications.[18–20]

The present study evaluated the effects of EMF on tooth movement in rats and determined significant differences between the tested groups. Tooth movement in SEMF group was significantly greater than that of Cg-Cnt but the largest extent of tooth movement was achieved in the PEMF group. Limited number of studies evaluated the effects of SEMF on tooth movement and contradictory results were also reported. Although Tengku et al. [21] reported that SEMF application did not enhance the orthodontic tooth movement, Sakata et al. [6] reported that the application of SEMF can accelerate the tooth movement in rats. On the other hand Showkatbakhsh et al. [22] reported that the accumulative toot movement was significantly larger in the PEMF group. Stark and Sinclair [23] reported that the rate of orthodontic tooth movement and bone deposition was increased after PEMF application. Similar results were obtained by Chen [24] who reported that the EMF could accelerate the rate of orthodontic tooth movement. On the other hand Darendeliler et al. [5] reported that under PEMF, the coil spring induced tooth movement at a significantly higher extent than that of coil–magnet combination. Although there were some differences in relation with the duration and the frequency of EMF applications, our results are in accordance with the results of several studies.[5,6,22–24]

EMF enhances DNA,[20] RNA [19] and protein production in cell cultures [18] and short-term EMF application is suggested to cause accelerated calcium uptake in cartilaginous embryonic chick limbs.[25] On the other hand studies which evaluated the effects of EMF on bone and cartilage reported that EMF increased the rates of cellular division and metabolism, and thus promoted increased healing of bony and cartilaginous defects.[26,27] Although the precise mechanism of accelerated tooth movement after EMF applications is unclear, the beneficial therapeutic and cellular effects are thought to be contributed to the process of orthodontic tooth movement.[5,23,28]

## Conclusions

Within the limitations, according to the results of the present study, the application of ELF-EMF accelerated the orthodontic tooth movement in rats. Due to the differences between body size, geometry and physiological responses extrapolation of these results to humans should not be straightforward, and any such comparison should be made with caution.
